# Analysis of Discordant Findings between 3T Magnetic Resonance Imaging and Arthroscopic Evaluation of the Knee Meniscus

**DOI:** 10.3390/jcm12175667

**Published:** 2023-08-31

**Authors:** Pieter Van Dyck, Jasper Vandenrijt, Thijs Vande Vyvere, Annemiek Snoeckx, Christiaan H. W. Heusdens

**Affiliations:** 1 Department of Radiology, Antwerp University Hospital, Drie Eikenstraat 655, 2650 Edegem, Belgium; thijs.vandevyvere@uantwerpen.be (T.V.V.); annemiek.snoeckx@uza.be (A.S.); 2 Faculty of Medicine and Health Sciences, University of Antwerp, Universiteitsplein 1, 2610 Wilrijk, Belgium; krik.heusdens@uza.be; 3 Department of Orthopedics, Antwerp University Hospital, Drie Eikenstraat 655, 2650 Edegem, Belgium; jasper.vandenrijt@uza.be

**Keywords:** knee injuries, magnetic resonance imaging, meniscus tear, accuracy, arthroscopy

## Abstract

Numerous studies have assessed the performance of magnetic resonance imaging (MRI) in detecting tears of the knee menisci using arthroscopy results as the gold standard, but few have concentrated on the nature of discordant findings. The purpose of this study was to analyze the discordances between 3T MRI and arthroscopic evaluation of the knee meniscus. Medical records of 112 patients who underwent 3T MRI and subsequent arthroscopy of the knee were retrospectively analyzed to determine the accuracy of diagnoses of meniscal tear. Compared with arthroscopy, there were 22 false-negative and 14 false-positive MR interpretations of meniscal tear occurring in 32 patients. Images with errors in diagnosis were retrospectively reviewed by two musculoskeletal radiologists in consensus and all errors were categorized as either unavoidable, equivocal or as interpretation error. Of 36 MR diagnostic errors, there were 16 (44%) unavoidable, 5 (14%) interpretation errors and 15 (42%) equivocal for meniscal tear. The largest categories of errors were unavoidable false-positive MRI diagnoses (71%) and equivocal false-negative MRI diagnoses (50%). All meniscal tears missed by MRI were treated with partial meniscectomy (n = 14) or meniscal repair (n = 8). Discordant findings between 3T MRI and arthroscopic evaluation of the knee meniscus remain a concern and primarily occur due to unavoidable and equivocal errors. Clinicians involved in the diagnosis and treatment of patients with meniscal tears should understand why and how the findings seen on knee MRI and arthroscopy may sometimes differ.

## 1. Introduction

Magnetic resonance imaging (MRI) is the keystone of musculoskeletal (MSK) imaging, including imaging of the knee meniscus [1,2]. Although arthroscopy remains the gold standard for diagnosing meniscal tears, MRI has become an increasingly used primary diagnostic tool in patients with knee symptoms due to its exquisite soft tissue contrast without using ionizing radiation and its lack of invasiveness [3,4].

The diagnostic performance of MRI compared to arthroscopy in detecting knee lesions is widely reported, and most studies have shown good performance for identifying meniscal tears [4,5,6]. However, there are far fewer studies available that performed an in-depth analysis of discordant findings between MRI and arthroscopic evaluation of the knee meniscus. Studies performed using 1.5T, dating back to the early 1990s [7,8], showed that most MRI diagnostic errors occurring at prospective readings also occur at retrospective readings, and thus can be called unavoidable. Errors encountered due to subtle MR findings that are equivocal for a tear are the second most common category, while interpretation (cognitive) errors are the least common [9,10]. A similar pattern of errors was found by Grossman et al. who used early generation 3T technology [11].

The field of MRI is evolving rapidly owing to technical advances, and knee imaging is certainly at the forefront of these developments [12,13]. In addition to the advent of clinical 3T systems in the early 2000s, other important steps that improved image quality in the years thereafter were the introduction of high-performance MRI gradient systems (comprising the second most expensive component of a scanner), advances in multichannel radiofrequency coil technology and the use of optimized pulse sequence designs [13].

As the findings seen on knee MRIs and arthroscopies may sometimes differ, it is important to understand the causes of errors [3]. Thus, the purpose of this study was to assess the frequency of discordant findings between current state-of-the-art 3T MRI and arthroscopic evaluation of the knee meniscus and to analyze the causes of the MRI diagnostic error. Based on previous studies, it was hypothesized that, despite recent technological developments over the last 15 years, the error patterns in the evaluation of the knee meniscus are similar to those reported in older studies.

## 2. Materials and Methods

Study participants for this single-center retrospective study were recruited from a patient cohort who underwent primary anterior cruciate ligament (ACL) reconstruction and/or meniscal surgery at our institution between January 2020 and December 2022. From this overall population, patients were eligible for participation in this study if they received a 3T knee MRI scan at our institution no longer than 80 days before surgery. All patients were included regardless of their age and chronicity of knee injury (MRI < 4 weeks from injury, acute and MRI > 4 weeks from injury, chronic). Patients with a history of meniscus and/or ACL surgery were also included. Patients with previous surgery other than meniscus or ACL were excluded to avoid artifacts of high-susceptibility metallic implants. Using these selection criteria, we identified a group of 112 subjects (mean age 39 years, range 9–71 years; female, n = 49; right knee, n = 57; mean time interval between MRI and arthroscopy 36 days, range 1–80 days). Ten (9%) patients previously underwent meniscus surgery, and four (4%) patients underwent prior ACL reconstruction. The study was approved by our institutional ethics committee and written informed consent was waived.

All arthroscopic meniscal evaluations were performed in our institution by one of three of our knee surgeons (each with at least 10 years of experience), who were aware of the prospective (original) MRI reports of the patients. Anterior arthroscopic portals were used in all patients; no posterior portals were used. In 112 patients, there were 64 tears of the medial meniscus (MM) and 41 tears of the lateral meniscus (LM) at arthroscopy. Sixty-nine (66%) of torn menisci were treated by partial meniscectomy and thirty-six (34%) of the torn menisci were repaired.

All study participants were imaged on a clinical whole-body 3T MRI scanner (Magnetom Skyra or PrismaFit; Siemens Healthcare) with a high-performance gradient system (45–80 mT/m gradient strength with a maximum slew rate of 200 T/m/s) using a dedicated 15-channel transmit–receive phased-array knee-coil (Quality Electrodynamics (QED), Mayfield Village, OH). The same routine MRI sequences were used for both 3T systems, with imaging parameters as follows: axial and coronal fat-saturated (FS) turbo spin-echo (TSE) proton density (PD)-weighted (TR/TE = 3560/28 ms, matrix 384 × 288, 3 mm slice-thickness-ST), sagittal TSE PD-weighted (TR/TE = 3000/21 ms, matrix 384 × 230, 2 mm ST), sagittal FS TSE T2-weighted (TR/TE = 3000/85 ms, matrix 384 × 230, 2 mm ST) and coronal TSE T1-weighted (TR/TE = 500/14 ms, matrix 384 × 216, 3 mm ST) with a FOV of 14–16 cm.

The original (prospective) MRI reports were generated by two separate board-certified MSK radiologists (each with more than 15 years of experience in MRI) in clinical routine using classic criteria of meniscus tear on MRI, including hyperintense signal abnormalities of the menisci definitely breaching the meniscal surface (grade 3), abnormal meniscal morphology, and displaced meniscal fragment [1,2]. For the post-operative meniscus, high T2-weighted signal reaching the meniscal surface or displaced meniscal fragment were considered a residual or recurrent tear [14]. Sixty-three tears of the MM and thirty-four tears of the LM were prospectively diagnosed at MRI.

The medical records and original MRI interpretations were retrospectively reviewed. The following information was retrieved from the arthroscopy reports: meniscal status (torn or intact) and, if present, location of the tear (anterior horn, body, posterior horn, or meniscus root). The type of meniscal tear was not recorded as knee surgeons at our hospital do not routinely specify the tear pattern. Based on the arthroscopic findings, accuracy of MRI for the MM was 79% with sensitivity and specificity of 81% and 77%, respectively (Table 1).

For the LM, MRI accuracy was 88% with sensitivity and specificity of 76% and 96%, respectively (Table 2).

All the cases with discordant results between MRI and operative findings were again assessed in consensus by the radiologists, as this gives an idea of the best possible result attainable, and then categorized as either unavoidable, equivocal or interpretation errors. Unavoidable errors were the MRI diagnostic errors that occurred at both prospective and retrospective assessments. Interpretation errors were those in which the original MR diagnosis differed from the surgical findings and the retrospective diagnosis of the readers. Equivocal errors occurred due to subtle MRI findings for a meniscal tear (e.g., a questionable articular surface or free-edge irregularity and increased intrameniscal signal that approaches the articular surface but does not definitely touch the surface, i.e., grade 2 meniscal signal).

## 3. Results

The 36 errors in diagnosis with MRI, including 22 false-negative and 14 false-positive MR interpretations of meniscal tear, had occurred in 32 patients (mean age 39 years, range 18–70 years; mean time interval between MRI and arthroscopy 32 days, range 1–80 days). Eighteen of these patients had an ACL rupture (acute, n = 16 and chronic, n = 2) and received an ACL reconstruction. Four patients had prior meniscus surgery and one patient had prior ACL reconstruction. Of the 36 errors, 16 (44%) were unavoidable errors, 5 (14%) were interpretation errors and 15 (42%) were equivocal errors. All meniscal tears missed by MRI were treated with partial meniscectomy (n = 14) or meniscal repair (n = 8). A detailed summary of the findings is given in Table 3.

### 3.1. Unavoidable Errors

**Figure 1:**
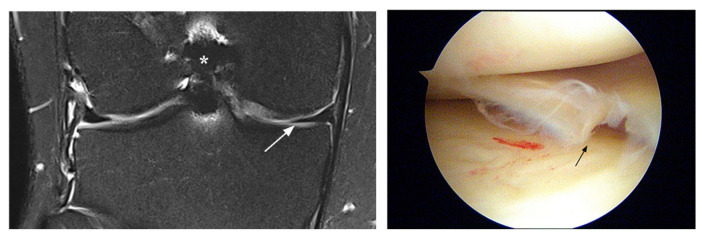
Unavoidable false-negative MRI diagnosis (patient 1). Coronal MRI shows normal appearance of the posterior horn of the medial meniscus (arrow), whereas the arthroscopic image indicates degenerative fraying with tear (arrow). Note status after ACL reconstruction (*).

**Figure 2:**
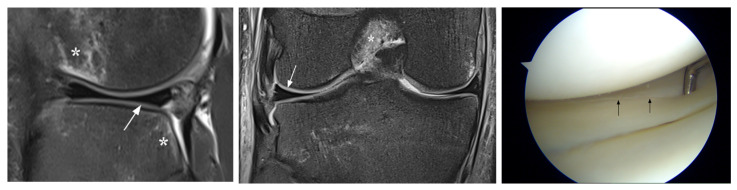
Unavoidable false-negative MRI diagnosis (patient 5). MRI shows normal appearance of the posterior horn of the lateral meniscus (arrows), whereas the arthroscopic image indicates an upper surface tear (arrows). Note bone contusions (*) at the lateral knee compartment due to acute ACL injury.

The unavoidable errors consisted of six false-negative and ten false-positive MR diagnoses of meniscal tear. Three false-negatives were in the posterior horn of the MM, with arthroscopic findings indicating degenerative fraying and tear (Figure 1) and three false-negatives were in the LM (anterior horn, n = 1 and posterior horn, n = 2), two of which had an associated acute ACL rupture (Figure 2). These six tears were diagnosed at arthroscopy but not seen on MRI, even in retrospect with knowledge of the tear location.

**Figure 3:**
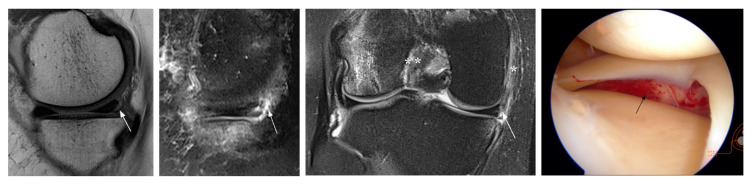
Unavoidable false-positive MRI diagnosis (patient 8). MRI shows unequivocal signs of a tear at the posteromedial meniscocapsular junction (arrows). Also note tearing at the medial collateral ligament (MCL, *) and acute ACL injury (**). Arthroscopy indicates a normal medial meniscus and hematoma at the MCL (arrow).

The 10 false-positive interpretations were all considered to exhibit unequivocal evidence of meniscal tear on MRI: 5 had MRI findings of a (meniscocapsular) tear of the posterior horn of the MM (Figure 3), 2 were apparent peripheral tears of the posterior horn of the LM, 1 was in the root of the posterior horn of the MM, and 2 post-operative MM were interpreted as torn on MRI by the radiologists but were not described by the arthroscopist. Of note, seven of the false-positive MR evaluations occurred in patients with an ACL-deficient knee.

### 3.2. Interpretation Errors

**Figure 4:**
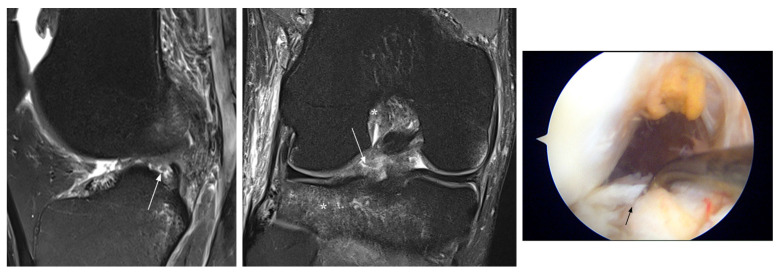
Interpretation false-negative MRI diagnosis (patient 18). MRI shows a tear at the posterolateral meniscal root that was missed at prospective reading and confirmed by arthroscopy (arrows). Also note lateral bone contusions and acute ACL injury (*).

**Figure 5:**
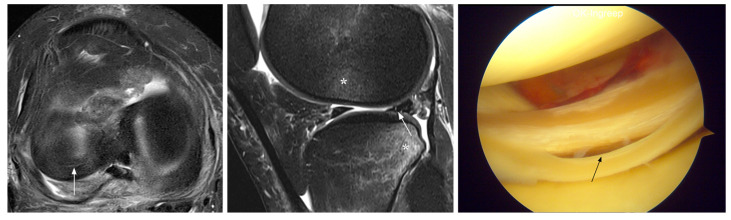
Interpretation false-negative MRI diagnosis (patient 19). MRI shows a tear at the posterior horn of the lateral meniscus that was missed at prospective reading and confirmed by arthroscopy (arrows). Also note lateral bone contusions due to acute ACL injury (*).

The interpretation errors consisted of five false-negative MR diagnoses of meniscal tear and all of them occurred in patients with an ACL rupture: three were posterior root tears (LM, n = 2 and MM, n = 1) (Figure 4) and two were peripheral tears of the posterior horn (MM, n = 1 and LM, n = 1) (Figure 5). Review of these missed tears revealed that these were visible on MRI retrospectively.

### 3.3. Equivocal Errors

**Figure 6:**
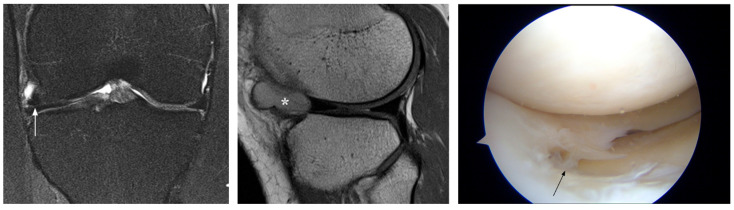
Equivocal false-negative MRI diagnosis (patient 27). MRI shows questionable grade 2 signal change at the anterior horn of the lateral meniscus (arrow) with a parameniscal cyst (*), but arthroscopy confirmed a degenerative tear at this location (arrow).

MR findings that were equivocal for meniscal tear caused 11 false-negative and 4 false-positive diagnoses, 7 of which occurred in patients with an ACL rupture. The largest category of errors was equivocal false-negative MRI diagnoses, including seven tears of the posterior horn of the MM (including one post-operative meniscus) and four tears of the LM (anterior horn, n = 2; body, n = 1 and posterior horn, n = 1). These menisci demonstrated equivocal findings for a meniscal tear on MRI, i.e., questionable grade 2 or 3 meniscal signal with or without contour abnormality), but arthroscopic findings indicated that the menisci were torn (Figure 6).

In the false-positive equivocal MRI cases, there were three apparent tears of the posterior horn of the MM (including one post-operative meniscus and one apparent ramp lesion in a patient with acute ACL rupture), and one apparent tear of the posterior horn of the LM.

## 4. Discussion

The main finding of the present study was that despite the use of modern 3T equipment, discordances between MRI and arthroscopic evaluation of the knee meniscus remain a concern in clinical practice. The most common errors in MRI interpretation were unavoidable errors (44%), occurring at both prospective and retrospective evaluation, and equivocal errors (42%), due to subtle findings that are equivocal for a meniscal tear. Interpretation errors (14%) were the least frequent.

Although the accuracy of MRI for diagnosing meniscal tears has been extensively reported in the literature [1,2,3,4,5,6], few studies have provided insights in the causes of discordances that may occur between MRI and arthroscopic evaluation of the menisci of the knee. Our results are in agreement with the earliest studies on the subject performed at 1.5T [7,8,10], and later on, at 3T [11], showing that most MRI diagnostic errors are unavoidable or due to equivocal findings. Compared to the study by Grossman et al. in 2009 [11], who used early 3T technology and an 8-channel phased-array knee coil, we used a powerful modern 3T machine equipped with an 18-channel knee coil. However, our results show that, despite the advances in MRI scanners and coil technology over the last 15 years, the frequency and nature of errors in the MRI evaluation of the knee meniscus are similar to those reported in older studies.

In our study, false-negative MR diagnoses outnumbered false-positive diagnoses. The largest category of false-negative diagnoses was due to equivocal MRI findings in the posterior horn of the medial meniscus, reflecting the problem of observer variation [15]. Typically, equivocal cases consist of menisci for which one or two images show questionable grade 2 signal changes or a grade 3 signal on a single image only [1,2]. Our findings contradict those of previous studies stating that a meniscal tear is unlikely when MR shows a focus of high signal intensity in the meniscus that does not unequivocally extend to involve the inferior or superior articular surface [9,16,17]. Furthermore, Elvenes et al. [18] stated that an important source of diagnostic errors is overcalling by radiologists, leading to false-positive MR results. Based on our study findings, we believe that radiologists should instead be descriptive in reporting subtle MR findings, alerting the clinician of possible meniscal tear. This is especially challenging in the menisci of older patients in which it may be unclear if MRI changes are the result of a tear or of age-related meniscal degeneration [19]. In agreement with these findings, we found patients in the equivocal group to be slightly older compared to those in the unavoidable group (mean age of 42 years vs. 37 years, respectively).

Unavoidable errors were the second most common category of false-negative MRI diagnoses, which illustrates the fundamental limits of spatial resolution in clinical knee MRI. Previous studies [7,8,10] found that many unavoidably missed tears on MRI are small and stable and do not require surgical treatment. However, in our study, all meniscal tears missed on MRI were treated surgically with partial meniscectomy or meniscal repair. Notably, in three unavoidable false-negatives in the posterior horn of the MM, the meniscal inner edge was said at arthroscopy to be frayed and torn, but the classification of these changes is subject to a wide range of interpretation by arthroscopists [7,8,10,19].

Although the readers in our study were experienced MSK radiologists, there were five false-negative MRI errors in patients with ACL rupture, including three meniscal root tears and two peripheral meniscal tears. This shows that a certain level of error is present in any level of experience. In addition, tear locations, such as the meniscal root insertions or the peripheral meniscocapsular portion, have been the most difficult for MRI to visualize adequately [2,20,21]. This can be explained by the fact that routine knee MRI protocols, even when performed at 3T, typically consist of separately acquired two-dimensional (2D) sequences, acquired with a large slice thickness (2–3 mm), which results in anisotropic voxel dimensions and partial volume averaging effects [2]. In contrast, 3D sequences provide a thin-partitioned single-slab isotropic volume covering the whole knee joint, thereby reducing partial volume effects and eliminating interslice gaps [13]. A major advantage of the 3D TSE sequences is that the source data can subsequently be reformatted in any desired orientation, which facilitates the depiction of oblique complex knee structures (e.g., meniscal roots and meniscocapsular attachments). Unfortunately, compared to 2D sequences, the 3D sequences have poor image quality and long acquisition times. Therefore, they are not routinely applied in clinical knee MRI protocols [12,13]. Recent developments in frontier technologies, including artificial intelligence, deep learning-based acquisition and image reconstruction techniques have shown tremendous potential for ultra-fast super-resolution MSK MRI, in the hope of facilitating the future use of 3D techniques in the clinic [12].

In our study, ten of the fourteen false-positive diagnoses were categorized as unavoidable. Of these, seven had definite MRI findings of tears at the medial meniscocapsular junction (n = 5) or posterior horn of the lateral meniscus (n = 2) in association with acute ACL rupture. One patient with chronic knee pain had MRI findings consistent with a tear of the posterior medial meniscus root, which was not confirmed during arthroscopy. According to previous studies, it is reasonable to argue that these false-positive MRI interpretations are related to problems with arthroscopy when using conventional anterior knee portals, including the lack of imaging of portions of the posterior horn of the MM and reliance on probing or compression to diagnose tears [3,22,23].

Our results are in agreement with previous studies reporting poorer performance of MRI to predict meniscal injuries present at acute ACL reconstruction [4,24]. In our study, meniscal tears were more likely to be missed on MRI in ACL-deficient knees compared to ACL-intact knees (13 vs. 9, respectively). However, in contrast to studies reporting lower sensitivity for detecting LM tears [20,24], we found the highest rates of false-negatives for MM tears, both in ACL-deficient and ACL-intact knees (missed MM and LM tears, 7 vs. 6 and 5 vs. 4, respectively). The reason for this dissimilarity remains unknown and further research in this regard is needed.

In our study, there were four diagnostic errors occurring in post-operative menisci, including two equivocal errors (one false-positive and one false-negative) and two unavoidable errors (two false-positives). This finding confirms the limitations of conventional MRI for the assessment of the post-operative meniscus, as changes in the appearance of the meniscus caused by surgery can either mimic or obscure recurrent or residual meniscus tears [14]. The two unavoidable false-positive cases (showing definite signs of remnant tear on MRI) demonstrate that arthroscopic probing is especially advantageous in assessing the integrity and stability of the post-operative meniscus.

The first limitation of this study is that it is prone to selection bias as only patients undergoing meniscus and/or ACL surgery were included. Second, although we reduced the risk of false-negative MRI by minimizing the time between MRI and surgery, it cannot be ruled out with complete certainty that a new injury was sustained. Similarly, with regard to the false-positive MRIs, it remains uncertain whether these represent true tears that were missed by arthroscopy or tears that healed before surgery. Third, due to the retrospective study design, we could not analyze the error patterns of the tear types as most meniscal tears were not described sufficiently in the arthroscopy report to allow characterization of the tear.

## 5. Conclusions

Despite continuous MRI technology developments over the last 15 years, discordant findings between 3T MRI and arthroscopic evaluation of the knee meniscus remain a concern in clinical practice, primarily due to unavoidable and equivocal errors. Clinicians involved in the diagnosis and treatment of patients with meniscal tears should understand why and how the findings seen on knee MRI and arthroscopy may sometimes differ.

## Figures and Tables

**Table 1:** MRI accuracy for medial meniscus tears.

MRI Diagnosis	Arthroscopic Diagnosis		
	Tear	No Tear	Total
Tear	52	11	63
No tear	12	37	49
Total	64	48	112

**Table 2:** MRI accuracy for lateral meniscus tears.

MRI Diagnosis	Arthroscopic Diagnosis		
	Tear	No Tear	Total
Tear	31	3	34
No tear	10	68	78
Total	41	71	112

**Table 3:** Categorization of discordances between MRI and arthroscopy.

Error Type		TI	MRI Findings	Arthroscopy Findings	ACL	Treatment
Unavoidable						
	FN MM					
1	M, 38 y	42	normal	degenerative lesion posterior horn	ACL-R	PM
2	M, 56 y	1	normal	fraying with tear posterior horn		PM
3	M, 57 y	35	normal	degenerative tear posterior horn		PM
	FN LM					
4	M, 27	47	normal	tear anterior horn		PM
5	M, 18	41	normal	upper surface tear posterior horn	acute	R
6	M, 18	25	normal	tear posterior horn	acute	PM
	FP MM					
7	F, 46 y	39	root tear posterior horn	normal		
8	F, 36 y	18	ramp lesion posterior horn	normal	acute	
6	M, 18 y	25	ramp lesion posterior horn	normal	acute	
9	M, 50 y	10	tear posterior horn PO remnant	intact stable PO remnant		
10	M, 25 y	11	ramp lesion posterior horn	normal	acute	
11	M, 34 y	8	ramp lesion posterior horn	normal	acute	
12	M, 25 y	24	ramp lesion posterior horn	normal	acute	
13	F, 40 y	50	tear posterior horn PO remnant	intact stable PO remnant		
	FP LM					
14	M, 28 y	8	peripheral tear posterior horn	normal	acute	
15	M, 61 y	80	peripheral tear posterior horn	normal	chronic	
Interpretation						
	FN MM					
16	M, 45 y	80	missed peripheral tear posterior horn	unstable tear posterior horn	chronic	R
17	M, 40 y	10	missed posterior root tear	oblique root tear	acute	R
	FN LM					
18	M, 48 y	50	missed posterior root tear	posterior root tear	acute	R
19	F, 41 y	9	missed tear posterior horn	unstable tear posterior horn	acute	PM
20	M, 36 y	30	missed posterior root tear	partial posterior root tear	acute	R
Equivocal						
	FN MM					
21	F, 56 y	5	grade 2 posterior horn	tear posterior horn		PM
14	M, 28 y	8	grade 2–3 posterior horn	undersurface tear posterior horn	acute	R
22	M, 40 y	74	grade 2 posterior horn	degenerative tear posterior horn		PM
23	M, 48 y	7	grade 2–3 posterior horn	large tear posterior horn	acute	PM
24	M, 31 y	7	grade 2 posterior horn	undersurface tear posterior horn	acute	R
25	F, 40 y	7	grade 2–3 posterior horn PO remnant	undersurface tear posterior horn PO remnant	acute	PM
26	F, 44 y	9	grade 2–3 posterior horn	undersurface tear posterior horn	acute	PM
	FN LM					
27	F, 32	47	grade 2 anterior horn with parameniscal cyst	tear anterior horn		PM
28	F, 52	34	grade 2 anterior horn	tear anterior horn		PM
29	F, 18	80	grade 2 posterior horn	undersurface tear posterior horn		R
10	M, 25	11	grade 2–3 posterior horn	small tear body posterior horn	acute	PM
	FP MM					
30	F, 51 y	27	grade 2–3 posterior horn PO remnant	intact PO remnant		
31	M, 70 y	70	grade 2–3 posterior horn	normal		
32	F, 24 y	23	equivocal ramp lesion posterior horn	normal	acute ACL	
	FP LM					
30	F, 51	27	grade 2 posterior horn	normal		

FP: false-positive; FN: false-negative; MM: medial meniscus; LM: lateral meniscus; PO: post-operative; ACL: anterior cruciate ligament; PM: partial meniscectomy; R: repair.

## Data Availability

The datasets analyzed during the current study are available from the corresponding author on reasonable request.
